# A tNGS-based comparative study on clinical features and co-detection profiles of hospitalized children with influenza A and B viruses

**DOI:** 10.3389/fmed.2026.1887047

**Published:** 2026-08-05

**Authors:** Chunyun Fu, Qiang Huang, Xiaoying Chen, Haifeng Huang, Junming Lu, Jia Lu, Jiaqi Wen, Lishai Mo, Wenting Tang, Jiangyang Zhao, Yili Zhang, Xiangjun Lu, Yanhua Feng, Yanqing Tang, Shang Yi, Hao Wei, Huiping Huang, Xuehua Hu, Jie Tan

**Affiliations:** 1 Medical Science Laboratory, Children's Hospital, Maternal and Child Health Hospital of Guangxi Zhuang Autonomous Region, Nanning, China; 2 The Second Clinical School of Shandong University, Jinan, China; 3 Department of Pediatric Respiratory Medicine, Children's Hospital, Maternal and Child Health Hospital of Guangxi Zhuang Autonomous Region, Nanning, China; 4 Department of Genetic Metabolism, Children's Hospital, Maternal and Child Health Hospital of Guangxi Zhuang Autonomous Region, Nanning, China

**Keywords:** co-detected, influenza B virus, pathogen, pediatric patients, tNGS

## Abstract

**Objectives:**

This study aimed to characterize the clinical and epidemiological features of hospitalized children with IBV and compare them with those of influenza A virus (IAV).

**Methods:**

We enrolled 10,143 pediatric inpatients with ARI between April 2021 and December 2024. Pathogen screening was conducted using targeted next-generation sequencing. Multivariable logistic regression was performed to identify independent predictors for respiratory support requirements.

**Results:**

IBV was detected in 198 children (2.0%). Compared to children with IAV (*n* = 325), IBV-positive children showed similar age distribution (median: 38 vs. 42 months, *p* = 0.464) and similarly high polymicrobial detection rates (96.0% vs. 97.2%, *p* = 0.365). The most frequently co-detected microbes were *Haemophilus influenzae*, *Streptococcus pneumoniae*, and *Mycoplasma pneumoniae*, similar between groups, though the prevalence of *H. influenzae* was higher in the IBV group than in the IAV group (*p* = 0.017). Children with IBV required less frequent respiratory support than those with IAV (12.6% vs. 20.6%, *p* = 0.020). However, multivariable analysis reversed this trend: IBV infection independently predicted a higher risk for respiratory support than IAV (aOR = 1.882, 95% CI: 1.134–3.123, *p* = 0.014). Younger age (aOR = 0.990, *p* = 0.009) and female sex (aOR for males = 0.503, *p* = 0.009) were also independent risk factors. Among IBV cases, 31.3% developed respiratory complications and 46.0% presented with extrapulmonary manifestations. Outcomes were favorable with a median hospitalization of 6 days, low ICU admission rate (4.0%), and no mortality.

**Conclusion:**

IBV and IAV show similar age distributions and comparably high polymicrobial detection rates in hospitalized children. Importantly, multivariable analysis unmasked that IBV infection independently predicted a higher requirement for respiratory support than IAV, with younger age and female sex identifying particularly vulnerable cohorts. Clinicians should maintain high vigilance for respiratory progression in pediatric IBV inpatients.

## Introduction

Influenza B virus (IBV) is a major respiratory pathogen that imposes a considerable disease burden on pediatric populations, accounting for a substantial proportion of influenza-related hospitalizations and contributing to significant morbidity during seasonal epidemics (1, 2). Although generally perceived as milder than influenza A virus (IAV) infections, IBV can cause severe complications, particularly in immunologically immature children (3). However, current understanding of the characteristics and clinical significance of co-detected microbial profiles in IBV infections remains limited. Conventional diagnostic methods (e.g., PCR or culture), with their restricted pathogen coverage, struggle to accurately characterize these complex microbial communities (4). Targeted next-generation sequencing (tNGS), leveraging its high sensitivity and broad microbe detection capacity, offers a novel approach to decipher the polymicrobial landscape of respiratory infections (5–7).

At present, systematic data on IBV infections—encompassing co-detected pathogen profiles, laboratory findings, imaging, and clinical features—remain scarce. To address this gap, this tNGS-based comparative study leveraged data from 10,143 children with acute respiratory infections to systematically evaluate and compare the clinical features and co-detection profiles of influenza A and B cases. By integrating multidimensional clinical and microbiological data, this research aims to establish a foundational understanding of the clinical context in which pediatric IBV infections occur.

## Materials and methods

### Study population

This retrospective cohort study analyzed data from 10,143 pediatric inpatients who underwent tNGS testing and met the criteria for acute respiratory tract infection (ARI) at the Maternal and Child Health Hospital of Guangxi Zhuang Autonomous Region between April 2021 and December 2024. Patient clinical information and laboratory results were extracted from the electronic medical record (EMR) system. ARI was defined as an acute illness (typically ≤21 days duration) characterized by new-onset respiratory symptoms attributable to infectious pathogens. Key presenting symptoms included cough, expectoration, dyspnea, pharyngitis, and rhinorrhea. Diagnostic categories encompassed acute upper respiratory infections, acute bronchitis, and community-acquired pneumonia (CAP).

### Inclusion and exclusion criteria

Inclusion required meeting all criteria:

1. Hospitalized children with acute respiratory infection
2. Age ≤18 years at admission
3. Available tNGS results

Exclusion applied for any of:

1. Missing critical clinical/laboratory data (demographics, tNGS reports, or diagnostic documentation)
2. Non-infectious respiratory inflammation (allergic, asthmatic, or irritant-induced)
3. Nosocomial infection onset

### Microbiological testing using targeted next-generation sequencing

Clinical specimens included 4,575 bronchoalveolar lavage fluid (BALF) samples, 4,642 oropharyngeal swabs, and 926 sputum samples, collected according to institutional protocols (8). Nucleic acid extraction was performed using the MagPure Pathogen DNA/RNA Kit (R6672-01B, Magen, Guangzhou, China). Libraries were prepared using the Respiration100™ multiplex PCR system (KingCreate, Guangzhou, China), which targets 65–198 common respiratory pathogens (see Supplementary Tables 1, 2), including IBV. Sequencing was performed on the Illumina MiniSeq platform.

Post-sequencing, raw data underwent quality control using FastQC v0.11.9, with the following criteria: (1) ≥ 85% of bases at Q30, (2) ambiguous base calls (N-content) < 0.1%, and (3) adapter contamination ≤5%. Adapter trimming and quality filtration were performed using fastp v0.20.1 (9), retaining reads >60 bp with ≥80% Q30 bases. Processed reads were mapped against the GRCh38 reference genome using Bowtie2 v2.4.1 (10). Genomic references were obtained from the NCBI Nucleotide database, RefSeq, and GenBank.

Microbial detection was determined based on the following thresholds: (i) For bacteria (excluding *Mycobacterium tuberculosis* complex), fungi, and atypical agents: amplicon coverage ≥50% and normalized read count ≥10; (ii) For viruses: amplicon coverage ≥50% with normalized read count ≥3, or normalized read count ≥10 alone; (iii) For *M. tuberculosis* complex: normalized read count ≥1. Detailed tNGS procedures have been described in our previous study (11).

### Clinical correlation assessment

Following bioinformatic analysis, all patient clinical records were independently reviewed by two infectious disease specialists to evaluate the clinical correlation of detected microorganisms. The assessment employed a comprehensive framework integrating four key dimensions:

1. Host Factors: Immunocompromised states (e.g., neutropenia, HIV, organ transplantation) or prematurity.
2. Microbiological Evidence: Microbial load from tNGS, results from conventional microbiology, and sample type (e.g., sterile vs. non-sterile sites).
3. Clinical & Radiological Presentation: Documented clinical history, presenting symptoms, radiographic features (e.g., new pulmonary infiltrates), and elevated inflammatory markers (e.g., CRP, PCT).
4. Epidemiological Context & Treatment Response: Community transmission patterns and observed response to targeted therapy.

In cases of discrepant assessments, a final determination was made by a senior attending physician. Following this comprehensive clinical adjudication, only isolates that achieved a final designation as “suspected pathogens” were advanced to the subsequent analytical phases, which encompassed epidemiological characterization, polymicrobial interaction assessments, and subgroup stratification. Conversely, microbial findings attributed to commensal colonization, procedural contamination, or the physiological respiratory microbiome were rigorously censored from the final analytic dataset to preserve the etiological fidelity of the study cohort.

### Statistical methods

All analyses were performed using IBM SPSS Statistics (v26.0). Continuous variables with normal distribution are presented as mean ± standard deviation (SD), with between-group comparisons analyzed by independent samples t-test. Non-normally distributed variables are reported as median (interquartile range, IQR) and compared using Mann–Whitney U tests. Categorical data are expressed as frequency counts (percentages), with group differences assessed by chi-square test. To identify independent predictors associated with the requirement for respiratory support and to control for potential clinical confounding factors, a multivariable logistic regression analysis was performed. Variables that were clinically relevant or recommended by screening [including influenza type (IBV vs. IAV, Ref: IAV), age (per month increase), sex (Male vs. Female, Ref: Female), and polymicrobial co-detection complexity (≥3 vs. <3 microbes, Ref: <3 microbes)] were entered simultaneously into the binary logistic regression model using the enter method. Adjusted odds ratios (aORs) and their 95% confidence intervals (CIs) were calculated. Statistical significance thresholds were established at *p* < 0.05 (significant) and *p* < 0.001 (highly significant).

## Results

### Detection rate

Within the cohort of 10,143 pediatric ARI inpatients, tNGS analysis identified IBV in 198 cases (overall prevalence: 2.0%). Stratification by specimen type showed distribution across 52 BALF samples (1.1%), 139 oropharyngeal swabs (3.0%), and 7 sputum specimens (0.8%). This distribution reveals significantly enhanced detection efficacy in oropharyngeal swabs compared to alternative specimen sources.

### Demographic and temporal characteristics of pediatric IBV cases

**Figure 1:**
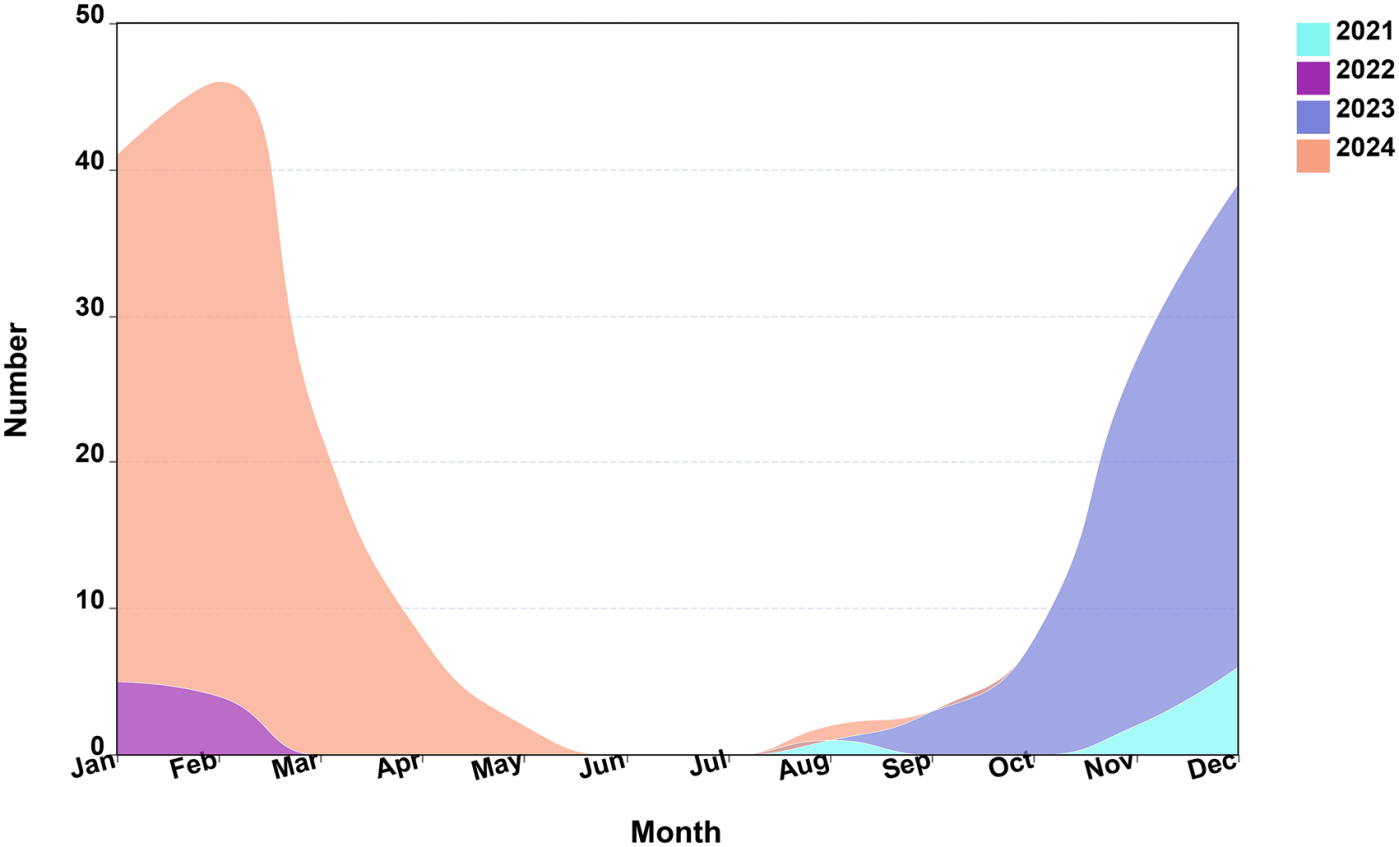
Monthly distribution of influenza B virus cases in pediatric respiratory hospitalizations.

The cohort of 198 IBV-detected children demonstrated a male predominance (127 males, 71 females; ratio 1.8:1) with ages spanning 1 month to 12 years. Age distribution was categorized into: <6 months (*n* = 19), 6–12 months (*n* = 34), 1–2 years (*n* = 22), 2–3 years (*n* = 17), 3–5 years (*n* = 51), and 5–12 years (*n* = 55). Clinical cases were principally managed across pediatric respiratory (57.1%), cardiovascular (17.2%), and infectious gastroenterology (15.2%) departments (Table 1). Temporal analysis identified a biennial surge in IBV detections during 2023–2024 versus 2021–2022, with consistent seasonal peaks occurring November–December through January–February annually (Figure 1).

**Table 1:** Characteristics of pediatric patients with the influenza B virus.

Characteristics	Count	Percentage
Age (*n* = 198)		
<6 months	19	9.6%
6 months-1 year	34	17.2%
1–2 years	22	11.1%
2–3 years	17	8.6%
3–5 years	51	25.8%
5–12 years	55	27.8%
Sex (*n* = 198)		
Male	127	64.1%
Female	71	35.9%
Department (*n* = 198)		
Pediatric Respiratory Medicine Department	113	57.1%
Department of Cardiology	34	17.2%
Department of Infectious and Digestive Diseases	30	15.2%
Pediatric Intensive Care Unit	8	4.0%
Department of Nephrology	7	3.5%
Department of Neurology	3	1.5%
Department of Hematology	1	0.5%
Department of Neonatology	1	0.5%
Department of Rehabilitation Medicine	1	0.5%

### Co-detection profiles in pediatric IBV cases

Microbiological assessment of 198 children with detected IBV revealed polymicrobial co-detections in 190 cases (96.0%), with only 8 cases (4.0%) showing IBV detection alone, underscoring the complex microbial ecology in these patients. Among cases with co-detections, the distribution of the number of detected microorganisms was as follows: a single co-detected microbe (*n* = 42, 22.1%), two (*n* = 49, 25.8%), three (*n* = 52, 27.4%), four (*n* = 32, 16.8%), and ≥5 (*n* = 15, 7.9%). Stratification by the composition of detected microbes identified two predominant patterns: (1) IBV-Bacterial-Viral co-detections (*n* = 66, 33.3% of the total cohort), characterized by the concurrent presence of respiratory viruses and bacteria alongside IBV; and (2) IBV-Bacterial co-detections (*n* = 58, 29.3% of the total cohort). The detailed distribution of co-detection patterns is presented in Table 2.

**Table 2:** Microbe co-detection patterns in hospitalized children with influenza B.

Microbial detection categories	Number (percentage)
IBV-Viral-bacterial co-detection	66 (33.3%)
IBV-Bacterial co-detection	58 (29.3%)
IBV-Viral-bacterial-atypical microbe co-detection	17 (8.6%)
IBV-Bacterial-atypical microbe co-detection	11 (5.6%)
Single IBV detection	8 (4.0%)
IBV-atypical microbe co-detection	7 (3.5%)
IBV-Viral co-detection	7 (3.5%)
IBV-Viral-atypical microbe co-detection	6 (3.0%)
IBV-Viral-bacterial-fungal co-detection	5 (2.5%)
IBV-Viral-fungal co-detection	3 (1.5%)
Other detection patterns	10 (5.1%)

IBV, influenza B virus.

### Spectrum of co-detected microorganisms

**Figure 2:**
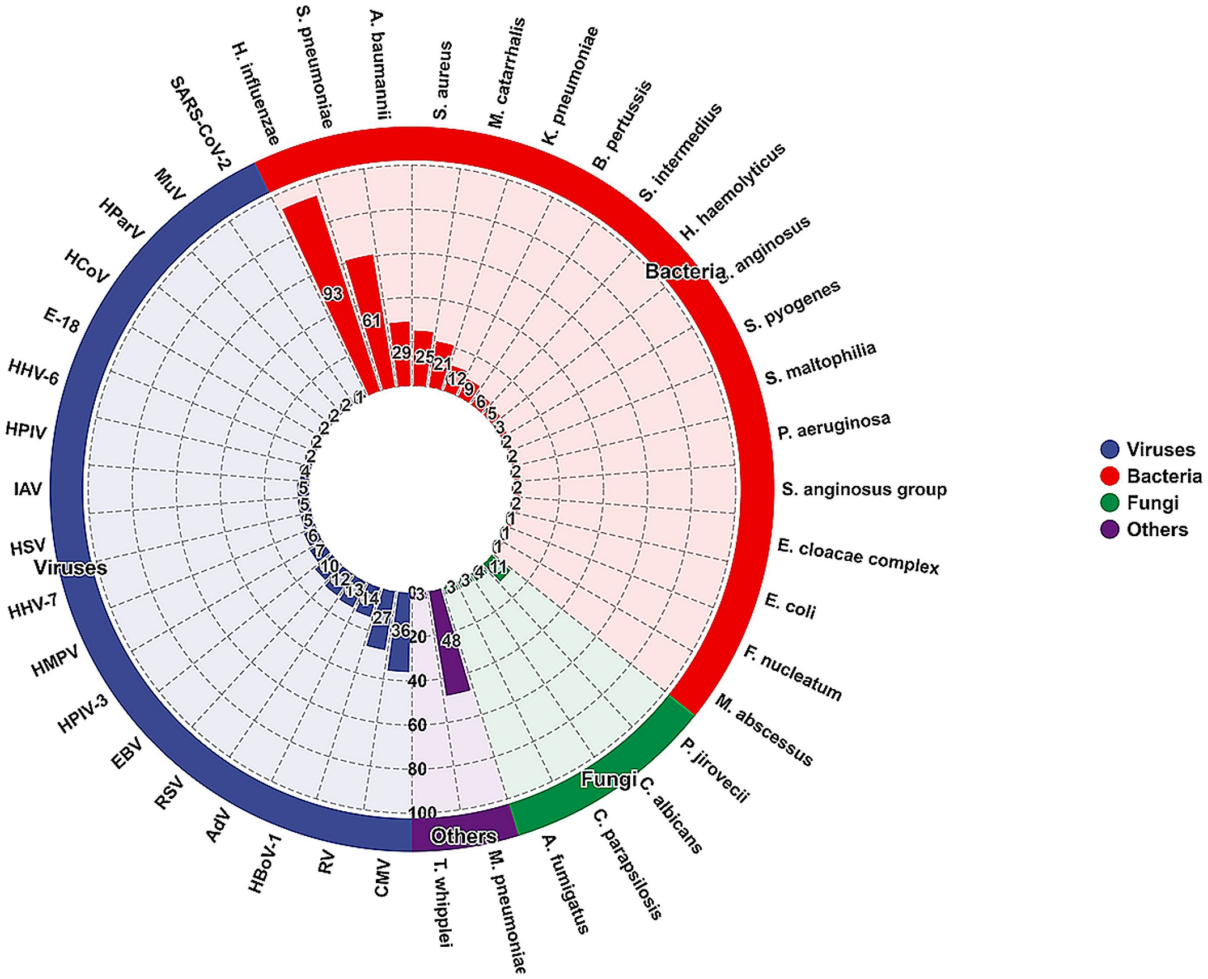
Distribution of co-detected microorganisms in children with influenza B virus. CMV: cytomegalovirus, RV: rhinovirus, RSV: respiratory syncytial virus, AdV: adenovirus, HHV-7: human herpesvirus 7, EBV: epstein–barr virus, HHV-6: human herpesvirus 6, HPIV-3: human parainfluenza virus 3, HMPV: human metapneumovirus, HSV: herpes simplex virus, IAV: influenza A virus, HPIV: human parainfluenza virus, HBoV-1: human bocavirus 1, E-18: echovirus E18, HCoV: human coronavirus, HParV: human parvovirus, MuV: human mumps virus, *H. influenzae*: *Haemophilus influenzae*, *S. pneumoniae*: *Streptococcus pneumoniae*, *A. baumannii*: *Acinetobacter baumannii*, *S. aureus*: *Staphylococcus aureus*, *M. catarrhalis*: *Moraxella catarrhalis*, *K. pneumoniae*: *Klebsiella pneumoniae*, *B. pertussis*: *Bordetella pertussis*, *S. intermedius*: *Streptococcus intermedius*, *H. haemolyticus*: Ha*emolytic Haemophilus*, *S. anginosus*: *Streptococcus anginosus*, *S. pyogenes*: *Streptococcus pyogenes*, *S. maltophilia*: *Stenotrophomonas maltophilia*, *P. aeruginosa*: *Pseudomonas aeruginosa*, *S. anginosus* gro*up*: *Streptococcus anginosus* gro*up*, *E. cloacae* com*plex*: *Enterobacter cloacae* com*plex*, *E. coli*: *Escherichia coli*, *F. nucleatum*: *Fusobacterium nucleatum*, *M. abscessus*: *Mycobacterium abscessus*, *P. jirovecii*: *Pneumocystis jirovecii*, *C. albicans*: *Candida albicans*, *C. parapsilosis*: *Candida parapsilosis*, *A. fumigatus*: *Aspergillus fumigatus*, *M. pneumoniae*: *Mycoplasma pneumoniae*, *T. whipplei*: *Tropheryma whipplei*.

Comprehensive tNGS profiling identified 42 distinct microbial species across the cohort of children with detected IBV. The six most frequently co-detected microorganisms comprised: *Haemophilus influenzae* (*n* = 93, 46.9%), *Streptococcus pneumoniae* (*n* = 61, 30.8%), *Mycoplasma pneumoniae* (*n* = 48, 24.2%), cytomegalovirus (*n* = 36, 18.2%), *Acinetobacter baumannii* (*n* = 29, 14.6%), and rhinovirus (*n* = 27, 13.6%). The full taxonomic distribution is visualized in Figure 2.

### Comparative analysis of clinical and laboratory features in children with detected IBV versus IAV

Among the 10,143 pediatric inpatients with ARI, IAV was detected by tNGS in 325 cases. Among these, IAV was detected alone in 9 cases (2.8% vs. IBV 4.0%, *p* = 0.418), while co-detection of other respiratory microorganisms was observed in 316 cases (97.2% vs. IBV 96.0%, *p* = 0.365). The predominant co-detection patterns included IAV-bacterial-viral (97 cases, 29.8% vs. IBV 34.7%, *p* = 0.226) and IAV-bacterial (91 cases, 28.0% vs. IBV 30.5%, *p* = 0.589). Microbiological profiling identified 41 co-detected microorganisms, with *Haemophilus influenzae* [37.2% (121/325) vs. 46.9% (93/198), *p* = 0.017], *Streptococcus pneumoniae* [30.8% (100/325) vs. 30.8% (61/198), *p* = 1.000], and *Mycoplasma pneumoniae* [21.2% (69/325) vs. 24.2% (48/198), *p* = 0.384] being the most frequently detected.

A comparison of clinical and laboratory findings between children with detected IBV and IAV is presented in Table 3. The IBV group showed a statistically significant association with a lower need for respiratory support (12.6% vs. 20.6%, *p* = 0.020). Several other parameters also demonstrated significant intergroup differences: compared to IAV group, IBV group had reduced leukocyte counts (median: 7.4 vs. 8.4 × 10^9^/L, *p* = 0.004), lower neutrophil percentages (52.8% vs. 59.7%, *p* = 0.024), decreased eosinophil proportions (0.3% vs. 0.5%, *p* = 0.007), elevated D-dimer levels (530 vs. 460 ng/mL FEU, *p* = 0.014), and reduced serum cystatin C concentrations (0.86 vs. 0.91 mg/L, *p* = 0.024). Notably, the median values for these laboratory metrics in both groups remained strictly within their respective normal clinical reference ranges, indicating that the clinical relevance of these statistically significant statistical differences at the cohort level remains uncertain and should be interpreted with caution.

**Table 3:** Clinical characteristics of pediatric patients with influenza B vs. influenza A detection.

Clinical features	IAV (*n* = 325)	IBV (*n* = 198)	*p* value
Quantitative data [M (IQR)]			
Age (months)	42 (18 ~ 67)	38 (13 ~ 65)	0.464
WBC (×10^9^ cells/L)	8.4 (6.2 ~ 11.3)	7.4 (5.6 ~ 10.7)	**0.004
NEU (%)	59.7 (46.7 ~ 72.8)	52.8 (40.7 ~ 64.4)	*0.024
Mon (%)	8.4 (6.7 ~ 11)	8.6 (6.7 ~ 10.9)	0.583
Eos (%)	0.5 (0.1 ~ 1.6)	0.3 (0.1 ~ 0.9)	**0.007
LYM (%)	28.9 (18.6 ~ 40.9)	35.2 (25 ~ 49.1)	0.402
RBC (×10^12^/L)	4.6 (4.3 ~ 4.8)	4.6 (4.23 ~ 4.9)	0.821
PLT (×10^9^/L)	317 (238 ~ 418)	297 (219 ~ 398)	0.089
CRP (mg/L)	6.67 (3.11 ~ 16.79)	5.15 (2.28 ~ 13.83)	0.050
PCT (ng/mL)	0.19 (0.1 ~ 0.43)	0.15 (0.09 ~ 0.51)	0.330
ESR (mm/h)	22 (13 ~ 36)	24 (14 ~ 40)	0.264
D-dimer (ng/mL FEU)	460 (320 ~ 770)	530 (350 ~ 810)	*0.014
LDH (U/L)	320 (273 ~ 365)	315 (269 ~ 385)	0.786
CK (U/L)	99 (63 ~ 163)	89 (56 ~ 124)	0.437
CK-MB (U/L)	22 (17 ~ 29)	21 (16 ~ 28)	0.376
AST (U/L)	37 (29 ~ 49)	35 (30 ~ 50)	0.210
ALT (U/L)	17 (12 ~ 23)	16 (11 ~ 22)	0.413
CYs-C (mg/L)	0.91 (0.79 ~ 1.06)	0.86 (0.77 ~ 1.01)	*0.024
Length of hospitalization (days)	6 (5 ~ 8)	6 (5 ~ 9)	0.298
Duration of fever (days)	2 (2 ~ 3)	3 (1 ~ 4)	0.180
Treatment expense (CNY)	6,459 (4,414 ~ 9,968)	6,836 (4,762 ~ 9,621)	0.625
Categorical data [*N* (%)]			
Gender (male)	195 (60.4%)	127 (64.8%)	0.314
Fever (yes)	211 (71%)	131 (73.2%)	0.615
ICU admission rate	15 (4.6%)	8 (4.0%)	0.756
Respiratory support	67 (20.6%)	25 (12.6%)	*0.020
Respiratory complications	108 (33.2%)	62 (31.3%)	0.650
Complications of other systems	134 (41.2%)	91 (46.0%)	0.289

*P < 0.05, **p < 0.01, and ***P < 0.001 represent statistically significant differences. IAV, influenza A virus; IBV, influenza B virus; WBC, White blood cell; NEU, neutrophil; LYM, lymphocyte, Mon, monocyte; Eos, eosinophil; Baso, basophil; RBC, red blood cell; PLT, platelet; CRP, C-reactive protein; PCT, procalcitonin; ESR, erythrocyte sedimentation rate; LDH, lactic acid dehydrogenase; CK, creatine kinase; CK-MB, creatine kinase-MB; AST, aspartate aminotransferase; ALT, Alanine Aminotransferase; CYs-C, cystatin C; CNY, Chinese Yuan; ICU, intensive care unit. The bold values indicate statistical significance (*P* < 0.05).

### Thoracic CT findings in children with detected IBV

Among 198 children with detected IBV, 138 underwent chest Computed Tomography (CT). The predominant abnormalities included: patchy infiltrates (*n* = 109, 79.0%) and bronchial wall thickening (*n* = 107, 77.5%). Consolidations affected 23 patients (16.7%) bilaterally and 24 (17.4%) unilaterally. Additional findings comprised mucous plugging (*n* = 15, 10.9%), ground-glass opacities (*n* = 14, 10.1%), and atelectasis (*n* = 3, 2.2%). Pleural abnormalities consisted of effusions (*n* = 10, 7.2%) and mediastinal lymphadenopathy (*n* = 10, 7.2%), while pleural thickening was uncommon (*n* = 3, 2.2%) (Supplementary Table 3). This constellation of features delineates the radiological spectrum of pulmonary manifestations in children with detected IBV.

### Respiratory support requirements and clinical complications

**Figure 3:**
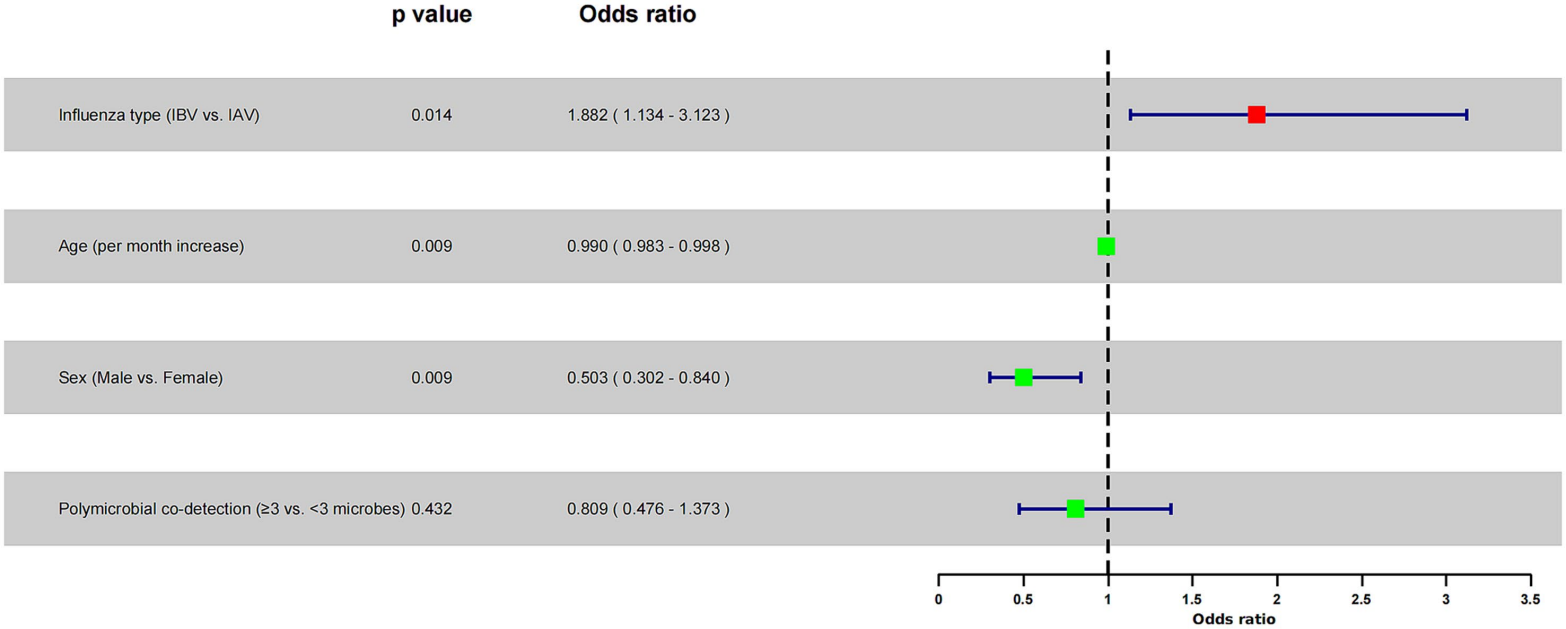
Forest plot of multivariable logistic regression analysis identifying independent predictors for requiring respiratory support. The dependent variable was the requirement for respiratory support. Variables entered into the model included influenza virus type (reference: IAV), age (per month increase), sex (reference: female), and polymicrobial co-detection complexity (reference: <3 microbes). Adjusted odds ratios (aORs) and their 95% confidence intervals (CIs) are presented. IBV infection, younger age, and female sex emerged as significant independent risk factors for an increased requirement for respiratory support. aOR, adjusted odds ratio; CI, confidence interval; IAV, influenza A virus; IBV, influenza B virus.

Among the 198 children with detected IBV, 25 (12.6%) received respiratory support. The interventions included low-flow oxygen therapy via nasal cannula or mask (*n* = 17, 8.6%), non-invasive ventilation (*n* = 1, 0.5%), and endotracheal intubation with ventilator support (*n* = 7, 3.5%). Notably, while the univariate analysis initially showed a lower crude rate of respiratory support in the IBV group compared to the IAV group (12.6% vs. 20.6%, *p* = 0.012), this trend was strikingly reversed after neutralizing baseline demographic and microbiological confounding factors. In the multivariable logistic regression model, IBV infection emerged as a significant independent risk factor for a higher requirement for respiratory support compared to IAV infection (aOR = 1.882, 95% CI: 1.134–3.123, *p* = 0.014; Figure 3). Additionally, younger age was identified as an independent risk factor for increased respiratory support, with the risk decreasing by 1% for each additional month of age (aOR = 0.990, 95% CI: 0.983–0.998, *p* = 0.009). In terms of sex, male patients showed a significantly lower risk of requiring respiratory support compared to female patients (aOR = 0.503, 95% CI: 0.302–0.840, *p* = 0.009). Polymicrobial co-detection complexity (≥3 vs. <3 microbes), however, did not show a statistically significant independent association (aOR = 0.809, 95% CI: 0.476–1.373, *p* = 0.432).

**Figure 4:**
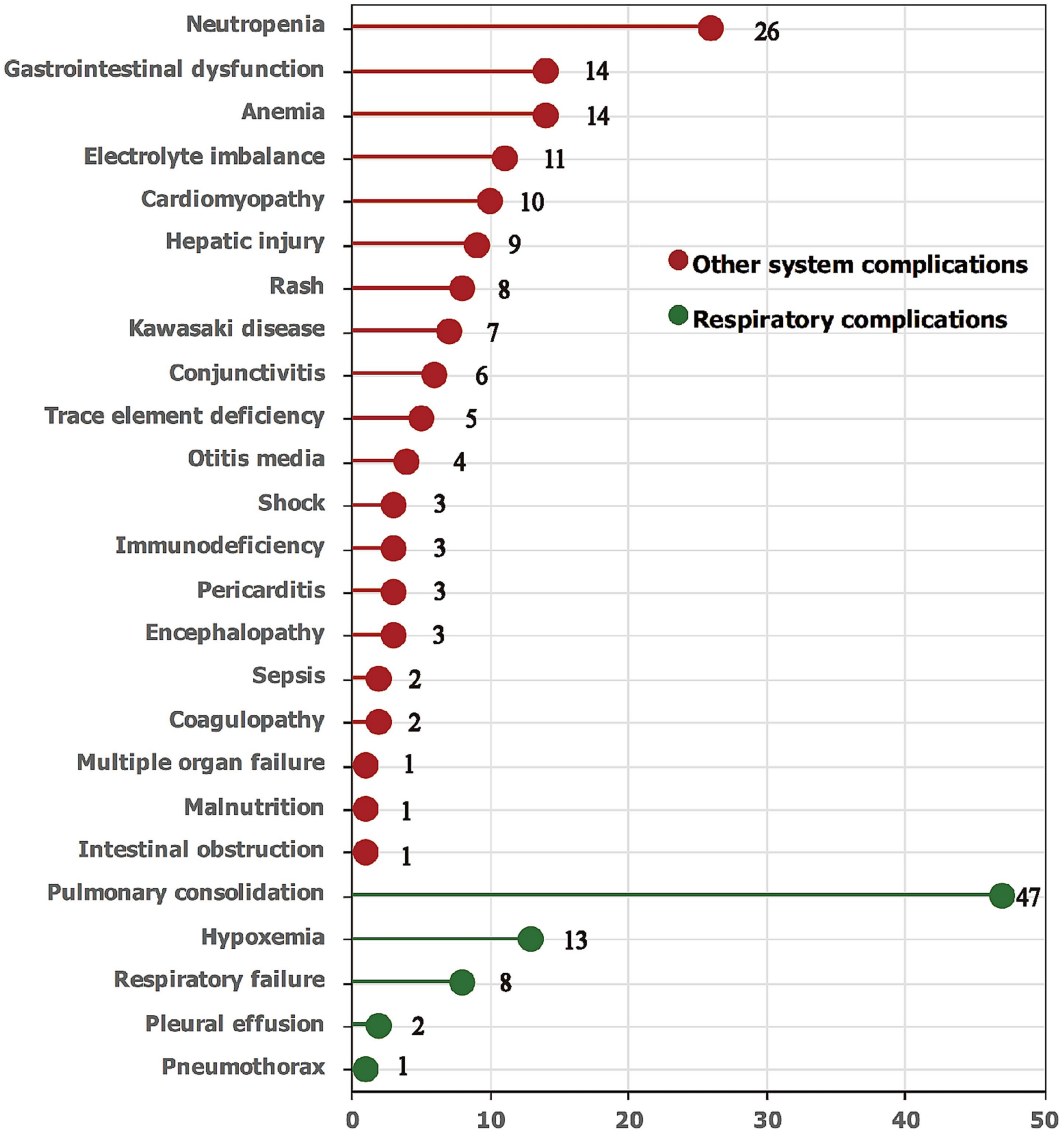
Clinical complications observed in children with detected influenza B virus.

Respiratory complications developed in 62 cases (31.3%), primarily pulmonary consolidation (*n* = 47) and hypoxemia (*n* = 13) (Figure 4). Other system complications affected 91 children (46.0%), with neutropenia (*n* = 26), gastrointestinal dysfunction (*n* = 14), and anemia (*n* = 14) representing the predominant manifestations (Figure 4).

### Clinical outcomes

Among the 198 children with detected IBV, 8 cases (4.0%) required intensive care unit (ICU) admission for management of critical illness, with a median hospitalization duration of 6 days (IQR: 5–9). Regarding clinical outcomes, 186 patients (93.9%) recovered and were discharged, while 12 cases (6.1%) were discharged upon parental request due to perceived suboptimal therapeutic efficacy or other reasons. No fatalities were recorded in this cohort.

## Discussion

Influenza B virus (IBV) is a well-recognized cause of pediatric respiratory infections worldwide, with reported detection rates of 3–9% in children (12–15). In this study, IBV was identified in 2.0% of hospitalized children with respiratory infections in Guangxi between 2021 and 2024. This relatively low detection rate is likely attributable to the concurrent COVID-19 pandemic and associated non-pharmaceutical interventions (NPIs), which suppressed influenza circulation in China before late 2022. Notably, the IBV positivity rate increased significantly during the 2023–2024 season following the relaxation of NPIs. Detection rates varied by specimen type: throat swabs (3.0%), bronchoalveolar lavage fluid (1.1%), and sputum (0.8%). However, specimen selection was clinically driven rather than randomized, introducing potential bias (16, 17). BALF is typically obtained from severely ill or complex cases, a population in whom prior antiviral use or lower viral loads in later disease stages may reduce detection rates (17). Thus, cross-specimen comparisons should be interpreted with caution. Among the 198 IBV-positive children, school-aged children (5–12 years) accounted for the largest proportion (27.8%), followed by infants <1 year (26.8%) and toddlers aged 3–5 years (25.8%). Infections peaked during the winter–spring season (November to February). These findings underscore the need for sustained IBV surveillance, particularly focusing on school-aged children and infants, to inform outbreak preparedness.

In this study, polymicrobial co-detections were observed in 96.0% of IBV-positive children, with over half involving three or more microorganisms. This high co-detection rate likely reflects the high sensitivity of tNGS and our targeted clinical cohort of hospitalized children with severe presentations (18). The most common co-detection profiles were IBV with bacteria and other viruses (33.3%) and IBV with bacteria (29.3%). Crucially, in the absence of an asymptomatic control group, these profiles must be interpreted with caution, as high co-detection rates do not inherently imply true pathogenic co-infection rather than microbial carriage. The high detection rates of microorganisms such as *Haemophilus influenzae* (46.9%), *Streptococcus pneumoniae* (30.8%), and *Mycoplasma pneumoniae* (24.2%) are consistent with some prior studies of respiratory infections (19, 20). Given that *H. influenzae* and *S. pneumoniae* are well-known common colonizers of the pediatric upper respiratory tract, their persistent detection by tNGS—even after our clinical filtering—likely reflects opportunistic carriage or proliferation enhanced by the primary viral infection, rather than acting as independent drivers of disease severity. Therefore, “microbial co-detection does not equal clinical co-infection.” Clinicians must meticulously synthesize tNGS data with the patient’s evolving clinical manifestations to avoid misinterpreting benign carriage as an active infection, thereby preventing unnecessary antimicrobial prescription and over-treatment. Future controlled studies are needed to clarify the potential interactions, if any, between IBV and these commonly detected microbes.

Despite recognized differences in clinical presentation between IBV and IAV infections, children with detected IBV and IAV in this study showed notable similarities in several metrics: comparable age distributions (median: 38 vs. 42 months, *p* = 0.464), nearly identical rates of polymicrobial co-detection (96.0% vs. 97.2%, *p* = 0.365), and the same three most frequently co-detected microorganisms—*Haemophilus influenzae*, *Streptococcus pneumoniae*, and *Mycoplasma pneumoniae*—although the prevalence of *Haemophilus influenzae* differed significantly (46.9% IBV vs. 37.2% IAV, *p* = 0.017). Additionally, no significant differences were found in hospitalization duration (median: 6 days both, *p* = 0.298), ICU admission rates (4.0% IBV vs. 4.6% IAV, *p* = 0.756), or treatment costs (CNY 6,836 IBV vs. 6,459 IAV, *p* = 0.625). The high degree of similarity in microbial co-detection profiles and healthcare resource utilization indicates that despite virological differences between IBV and IAV, they resulted in comparable clinical impact and healthcare burden in this hospitalized pediatric cohort. Furthermore, regarding the statistically significant differences observed in certain laboratory parameters (WBC, neutrophil%, eosinophil%, D-dimer, and cystatin C), their median values consistently fell within standard clinical limits. Consequently, these minor statistical variations between the two groups lack definitive clinical distinctiveness and likely reflect subtle systemic fluctuations rather than divergent disease mechanisms, further supporting the baseline clinical equivalence of IBV and IAV presentation in this cohort.

However, relying solely on crude univariate comparisons can mask the true pathogenic impact of specific viruses due to baseline clinical confounding. A critical and intriguing finding of our study emerged when we adjusted for age, sex, and microbial co-detection complexity using a multivariable logistic regression model. Interestingly, a “reversal effect” (Simpson’s Paradox) was observed: while the crude respiratory support rate was lower in the IBV group (12.6% vs. 20.6%), the multivariable model revealed that IBV infection was actually a significant independent predictor for a higher requirement for respiratory support compared to IAV (aOR = 1.882, 95% CI: 1.134–3.123, *p* = 0.014; Figure 4).

This independent association suggests that when comparing pediatric patients with identical age and demographic profiles, IBV carries an inherently substantial—and potentially underestimated—risk for severe respiratory compromise. This challenges the traditional clinical perception that IBV infections are universally milder than IAV. Furthermore, consistent with pediatric clinical experience, younger age (aOR = 0.990, *p* = 0.009) was confirmed as an independent risk factor for respiratory intervention, underscoring the anatomical vulnerability of infants and young toddlers. Interestingly, the model also revealed that female patients faced a significantly higher independent risk of requiring respiratory support compared to male patients (aOR = 0.503 for males, *p* = 0.009), suggesting potential sex-specific differences in immune responses or clinical presentations in this hospitalized cohort that warrant further investigation. These findings suggest that clinicians should maintain high vigilance for severe respiratory progression in children hospitalized with IBV, particularly in younger and female cohorts, rather than assuming a benign course relative to IAV.

The clinical outcomes observed in this cohort of children with detected IBV provide a descriptive overview of their management course. The relatively low requirement for respiratory support (12.6%), predominantly conventional oxygen therapy, was noted. However, the small subset requiring mechanical ventilation (3.5%) indicates that severe respiratory progression can occur, particularly in children with specific risk factors. Respiratory manifestations, including pulmonary consolidation and hypoxemia, were documented in 31.3% of cases, consistent with the known potential of influenza viruses to cause lower respiratory tract involvement (21, 22). Extrapulmonary manifestations were identified in 46.0% of children, with neutropenia and gastrointestinal disturbances being the most common, highlighting that systemic clinical features can accompany IBV detection. While no fatalities occurred and most patients (93.9%) recovered, the 4.0% ICU admission rate and cases of discharge upon parental request indicate that the illness course can be substantial in a minority. These findings collectively suggest that the management of children with detected IBV should account for the potential of severe respiratory progression and systemic manifestations, even in the absence of a high mortality risk.

This study has several limitations. First, the potential influence of underlying comorbidities on disease severity or prognosis could not be fully excluded. Second, the specific role of each co-detected microorganism in the infection process remains unclear, including potential synergistic or antagonistic effects among them. Third, as a retrospective, single-center study, the findings may be affected by selection bias, information bias, and other confounding factors. Therefore, the results should be validated in multicenter, prospective studies.

In conclusion, IBV was detected in 2.0% of hospitalized children with respiratory infections in Guangxi, with winter–spring seasonality and a bimodal age peak in school-aged children and infants. While polymicrobial co-detections were common (96.0%), their clinical significance remains unclear without asymptomatic controls. Although outcomes were generally favorable, multivariable analysis revealed that IBV infection independently predicted a higher requirement for respiratory support compared to IAV, with younger age and female sex serving as additional independent risk factors. Sustained surveillance and prospective controlled studies are warranted.

## Data Availability

The raw data supporting the conclusions of this article will be made available by the authors, without undue reservation.
