# Supplementary material for: A tNGS-based comparative study on clinical features and co-detection profiles of hospitalized children with influenza A and B viruses
Source: Front Med (Lausanne). 2026 Aug 5;13:1887047. doi: 10.3389/fmed.2026.1887047 (PMC13486149; doi:10.3389/fmed.2026.1887047)
Supplement: Supplementary file 1 [file Table_1.DOCX]

**Supplementary Table 1**. 65 respiratory microbes (including 39 viruses, 18 bacteria, 4 fungi, and 4 atypical microbes) detected by tNGS.

| **Microbial types** | **Microbes** |
| --- | --- |
| **Viruses (39)** | Influenza A virus, Influenza A virus H1N1, Influenza A virus H3N2, Influenza A virus H5N1, Influenza A virus H7N9, Mumps virus, Parvovirus B-19, Influenza A virus H1N1 (2009), Adenovirus Group B, Adenovirus Group C, Adenovirus Group E, Rubella virus, Influenza B virus, Measles virus, Influenza C virus, Rotavirus, Human Bocavirus 1, Enterovirus, Human Bocavirus 4, Enterovirus 71, Human Metapneumovirus, Rhinovirus A, Coronavirus 229E, Rhinovirus B, Coronavirus HKU1, Rhinovirus C, Coronavirus NL63, Herpes Simplex Virus Type 1 (HSV-1), Coronavirus OC43, Herpes Simplex Virus Type 2 (HSV-2), Parainfluenza virus Type 1, Varicella-zoster virus, Parainfluenza virus Type 2, Epstein-Barr virus, Parainfluenza virus Type 3, Cytomegalovirus, Parainfluenza virus Type 4, Respiratory syncytial virus A (RSV-A), Respiratory syncytial virus B (RSV-B). |
| **Bacteria (18)** | Streptococcus pneumoniae, Serratia marcescens, Corynebacterium diphtheriae, Haemophilus influenzae, Streptococcus pyogenes, Klebsiella oxytoca, Moraxella catarrhalis, Legionella pneumophila  Stenotrophomonas maltophilia, Enterococcus faecalis, Enterococcus faecium, Bordetella pertussis, Mycobacterium tuberculosis complex, Nontuberculous mycobacteria, Klebsiella pneumoniae, Staphylococcus aureus, Acinetobacter baumannii, Pseudomonas aeruginosa. |
| **Fungi (4)** | Cryptococcus neoformans, Aspergillus fumigatus, Pneumocystis jirovecii, Candida albicans. |
| **Atypical microbes (4)** | Chlamydia pneumoniae, Mycoplasma pneumoniae, Chlamydia psittaci, Chlamydia trachomatis. |

**Supplementary Table 2**. 198 respiratory microbes (including 80 bacteria, 79 viruses, 32 fungi, and 7 atypical microbes) detected by tNGS.

| **Microbial types** | **Microbes** |
| --- | --- |
| **Viruses (79)** | BK polyomavirus (human polyomavirus type 1), human adenovirus type 11, human respiratory syncytial virus type A, human adenovirus type 1, human adenovirus type 14, human respiratory syncytial virus type B, human adenovirus type 2, human adenovirus type 34, human rhinovirus type 1 (human parainfluenza virus type 1), human adenovirus type 21, human adenovirus type 35, human rhinovirus type 3 (human parainfluenza virus type 3), human adenovirus type 5, human herpesvirus 6A, influenza A virus, human adenovirus type 55, human herpesvirus 6B, influenza A virus H1N1, human adenovirus type 57, JC polyomavirus (human polyomavirus type 2), influenza A virus H3N2, human adenovirus type 6, WU polyomavirus (human polyomavirus type 4), influenza A virus H5N1, human adenovirus type 7, Coxsackievirus A5, influenza A virus H7N9, human adenovirus type 3, Coxsackievirus A6, influenza B virus, human adenovirus type 4, Coxsackievirus B3, Coxsackievirus A10, human herpesvirus type 1 (HSV1), Echovirus E18, Coxsackievirus A16, human herpesvirus type 2 (HSV2), Echovirus E30, Coxsackievirus A2, human herpesvirus type 3 (VZV), enterovirus, influenza C virus, human herpesvirus type 5 (CMV), enterovirus group A, influenza A virus H1N1 (2009), human herpesvirus type 6, enterovirus A71, influenza B virus Victoria lineage, human herpesvirus type 7, enterovirus group B, influenza B virus Yamagata lineage, human bocavirus type 1, enterovirus group C, measles virus, human bocavirus type 2, enterovirus group D, mumps virus, human bocavirus type 3, enterovirus D68, rhinovirus, human bocavirus type 4, human coronavirus 229E, rhinovirus type A, human herpesvirus type 4 (EBV), human coronavirus HKU1, rhinovirus type B, human adenovirus, human coronavirus NL63, rhinovirus type C, human adenovirus group B, human coronavirus OC43, rubella virus, human adenovirus group C, human metapneumovirus, SARS-CoV-2 , human adenovirus group D, human parainfluenza virus type 2 (human mumps virus type 2), human parvovirus B19, human parainfluenza virus type 4 (human mumps virus type 4). |
| **Bacterium (80)** | Corynebacterium diphtheriae, Nocardia saintgeorgensis (Nocardia gelsenkirchenensis), Klebsiella aerogenes, Mycobacterium asiaticum, Nocardia farcinica, Klebsiella oxytoca, Mycobacterium avium, Nocardia nova, Klebsiella pneumoniae, Mycobacterium avium complex (MAC), Nocardia otitidiscaviarum, Klebsiella variicola, Mycobacterium occultum, Nocardia terpeneae, Legionella spp., Mycobacterium gordonae, Micromonospora parva, Legionella bozemanii, Mycobacterium intracellulare, Rhodococcus equi, Legionella pneumophila, Mycobacterium kansasii, Staphylococcus aureus, Legionella longbeachae, Mycobacterium malmoense, Streptococcus agalactiae, Legionella micdadei, nontuberculous mycobacteria (NTM), Streptococcus anginosus group, Moraxella catarrhalis, Mycobacterium scrofulaceum, Streptococcus intermedius, Acinetobacter baumannii, Mycobacterium schroeteri, Streptococcus pneumoniae, Acinetobacter johnsonii, Mycobacterium simiae, Streptococcus pyogenes, Acinetobacter ursingii, Mycobacterium szulgai, Tropheryma whipplei, Bacteroides fragilis, Mycobacterium tuberculosis complex, Peptostreptococcus anaerobius, Homburgeria, Mycobacterium ranarum, Burkholderia cepacia complex, Bordetella parapertussis, Mycobacterium abscessus, Burkholderia mallei, Bordetella pertussis, Mycobacterium chelonae-abscessus complex, Burkholderia pseudomallei, Brucella spp., Mycobacterium chelonae, Burkholderia contaminans, Burkholderia cepacia nova, Mycobacterium fortuitum, Burkholderia multivorans, Burkholderia cepacia, Mycobacterium smegmatis, Elizabethkingia anophelis, Neisseria meningitidis, Nocardia spp., Elizabethkingia meningoseptica, Pasteurella multocida, Nocardia abscessus, Enterobacter cloacae complex, Proteus mirabilis, Nocardia africana, Escherichia coli, Pseudomonas aeruginosa, Nocardia asteroides, Fusobacterium necrophorum, Serratia marcescens, Nocardia brasiliensis, Fusobacterium nucleatum, Stenotrophomonas maltophilia, Nocardia cavae, Haemophilus influenzae. |
| **Fungi (32)** | Candida glabrata, Fusarium spp., Rhizomucor pusillus, Aspergillus flavus complex, Histoplasma capsulatum, Rhizopus spp., Aspergillus fumigatus, Mucorales spp., Rhizopus delemar, Aspergillus niger complex, Mucor plumbeus, Rhizopus microsporus, Aspergillus terreus complex, Mucor ramosissimus, Rhizopus oryzae, Candida albicans, Candida guilliermondii (Pichia guilliermondii), Cephalosporium spp., Candida pseudoglabrata, Mucor irregularis, Cephalosporium acremonium, Candida parapsilosis, Mucor racemosus, Cephalosporium boydii, Candida tropicalis, Pichia kudriavzevii (Candida krusei), Talaromyces marneffei, Cryptococcus gattii, Pneumocystis jirovecii, Trichosporon asahii, Cryptococcus neoformans, Mucor rhizopus. |
| **Atypical microbes (7)** | Chlamydia pneumoniae, Mycoplasma pneumoniae, Coxiella burnetii, Chlamydia psittaci, Ureaplasma parvum, Chlamydia trachomatis, Ureaplasma urealyticum. |

**Supplementary Table 3.** Thoracic CT Findings in Hospitalized Children with Detected Influenza B Virus (N = 138).

| **CT Imaging Abnormalities** | **Number of Patients (n)** | **Percentage (%)** |
| --- | --- | --- |
| **Pulmonary Infiltrates & Airway Changes** |  |  |
| Patchy infiltrates | 109 | 79.0% |
| Bronchial wall thickening | 107 | 77.5% |
| Mucous plugging | 15 | 10.9% |
| Ground-glass opacities | 14 | 10.1% |
| Atelectasis | 3 | 2.2% |
| **Consolidation** |  |  |
| Unilateral consolidation | 24 | 17.4% |
| Bilateral consolidation | 23 | 16.7% |
| **Pleural & Mediastinal Abnormalities** |  |  |
| Pleural effusions | 10 | 7.2% |
| Mediastinal lymphadenopathy | 10 | 7.2% |
| Pleural thickening | 3 | 2.2% |
